# Combined bidirectional spinal cord stimulation for refractory neuropathic pelvic pain: a case report

**DOI:** 10.3389/fpain.2026.1864829

**Published:** 2026-07-01

**Authors:** Yakov Perper, Alana Taub, Jenifer Perper

**Affiliations:** 1 Department of Anesthesiology, Mount Sinai Hospital of Queens, Queens, NY, United States; 2 New York Institute of Technology College of Osteopathic Medicine, Old Westbury, NY, United States

**Keywords:** combined bidirectional, lead blank, pelvic pain, spinal cord stimulator, subdermal security loop

## Abstract

**Introduction:**

Refractory neuropathic pelvic pain significantly impairs quality of life and is notoriously difficult to treat. We present the case of a 63-year-old woman who developed severe pelvic pain following emergency open thoracic aortic aneurysm repair. Despite extensive pharmacologic, non-pharmacologic, and interventional treatments, she experienced minimal relief.

**Methods:**

To address her pain, we attempted a novel approach termed “Combined Bidirectional”: anterograde (thoracic) and retrograde (sacral) spinal cord stimulator (SCS) placement, accompanied by combination therapy—high-frequency stimulation (HFX) at the thoracic spine and tonic stimulation at the sacral spine.

**Case description:**

Technical difficulties complicated retrograde sacral placement, and we discussed our approach to managing these challenges. Additionally, the patient developed unexpected hip pain with tonic sacral stimulation, prompting discontinuation of this modality and continuation of HFX thoracic stimulation alone.

**Conclusion:**

This case underscores both the potential advantages and challenges of combined bidirectional SCS for pelvic pain. Further research is warranted to clarify the efficacy and optimal application of this novel approach for refractory neuropathic pelvic pain.

## 1 Introduction

Chronic pelvic pain affects 15% to 25% of women (1) and an increasing number of men, with prevalence rates like those of migraines or lower back pain. The persistent, treatment-resistant nature of chronic neuropathic pelvic pain often leads to profound, multidimensional impairment—negatively impacting physical mobility, psychological health, and both social and intimate relationships. Because this condition frequently resists conventional multimodal therapies, patients are at risk of experiencing a sense of clinical abandonment. Spinal cord stimulator (SCS) has therefore emerged as a promising intervention for individuals with refractory neuropathic pelvic pain, particularly when standard treatments are ineffective. Recent evidence supports the efficacy of combined SCS approaches involving both anterograde and retrograde lead placements (2, 3). Furthermore, ongoing research into novel SCS combination therapies continues to broaden the potential applications and efficacy of this modality (4–6). The use of bidirectional lead placement—caudocephalad for thoracic and cephalocaudal for sacral regions—combined with combination therapy that stimulates distinct spinal segments using different modes, offers a logical and promising strategy. This case report details the application of this approach.

## 2 Case description

We present the case of a 63-year-old woman with normal BMI and no significant past medical history other than hypertension. She developed severe, burning neuropathic pelvic pain after an emergency open thoracic aortic aneurysm repair performed in the summer of 2021. The pain began two weeks later and severely impacted her well-being. Even basic tasks, such as personal hygiene, were difficult. She underwent many treatments: pelvic floor physical therapy, psychotherapy, pudendal nerve blocks, ganglion impar blocks, caudal epidural steroid injection, and bilateral S1 transforaminal epidural steroid injections, but had minimal relief. Multiple medications were tried (gabapentinoids, duloxetine, tramadol, and medical marijuana), all with little benefit. We discussed the potential benefits and limitations of stimulating two separate regions of the spinal cord simultaneously to address her pelvic pain. All questions and concerns were answered prior to obtaining informed consent.

**Figure 1:**
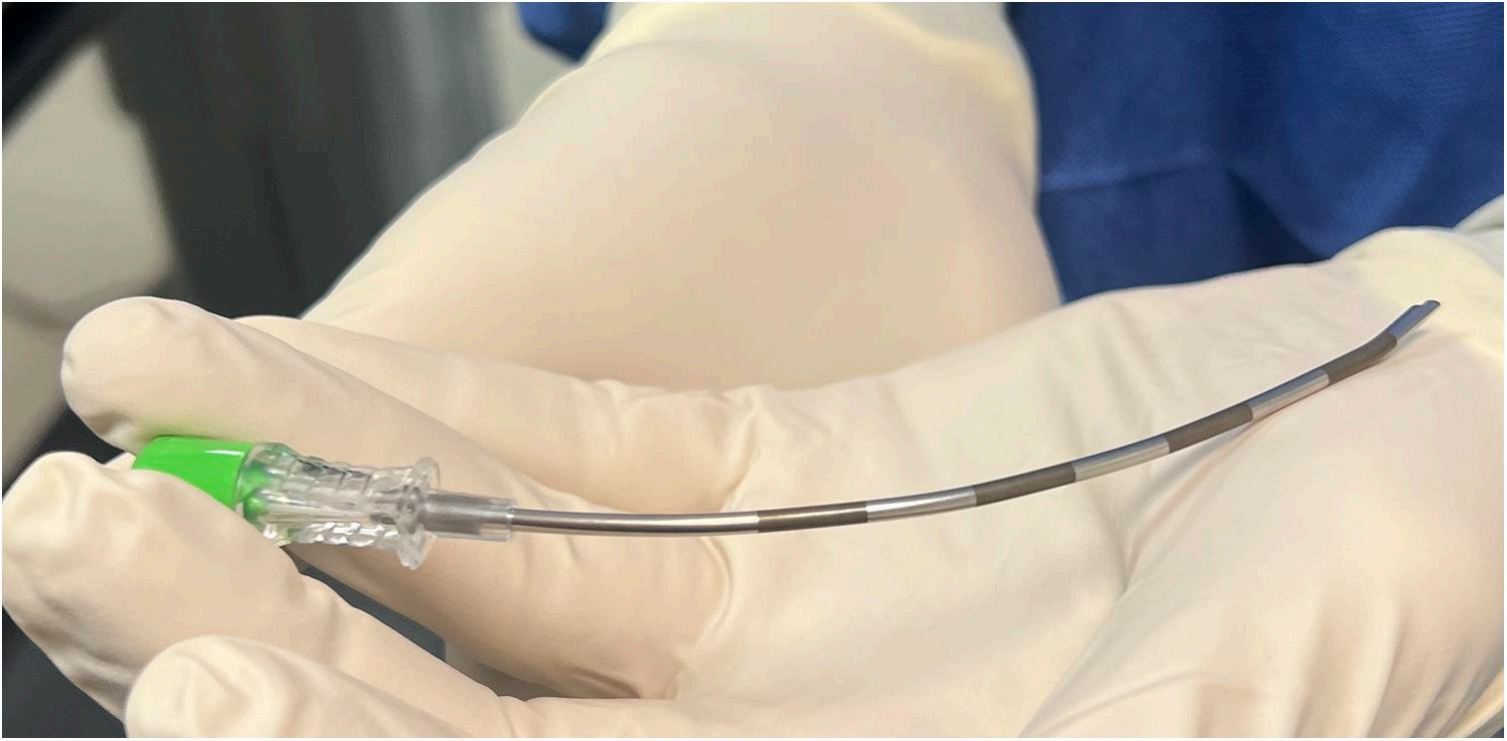
The 14 G epimed needle was bent in the opposite direction of the bevel opening to facilitate retrograde placement.

**Figure 2:**
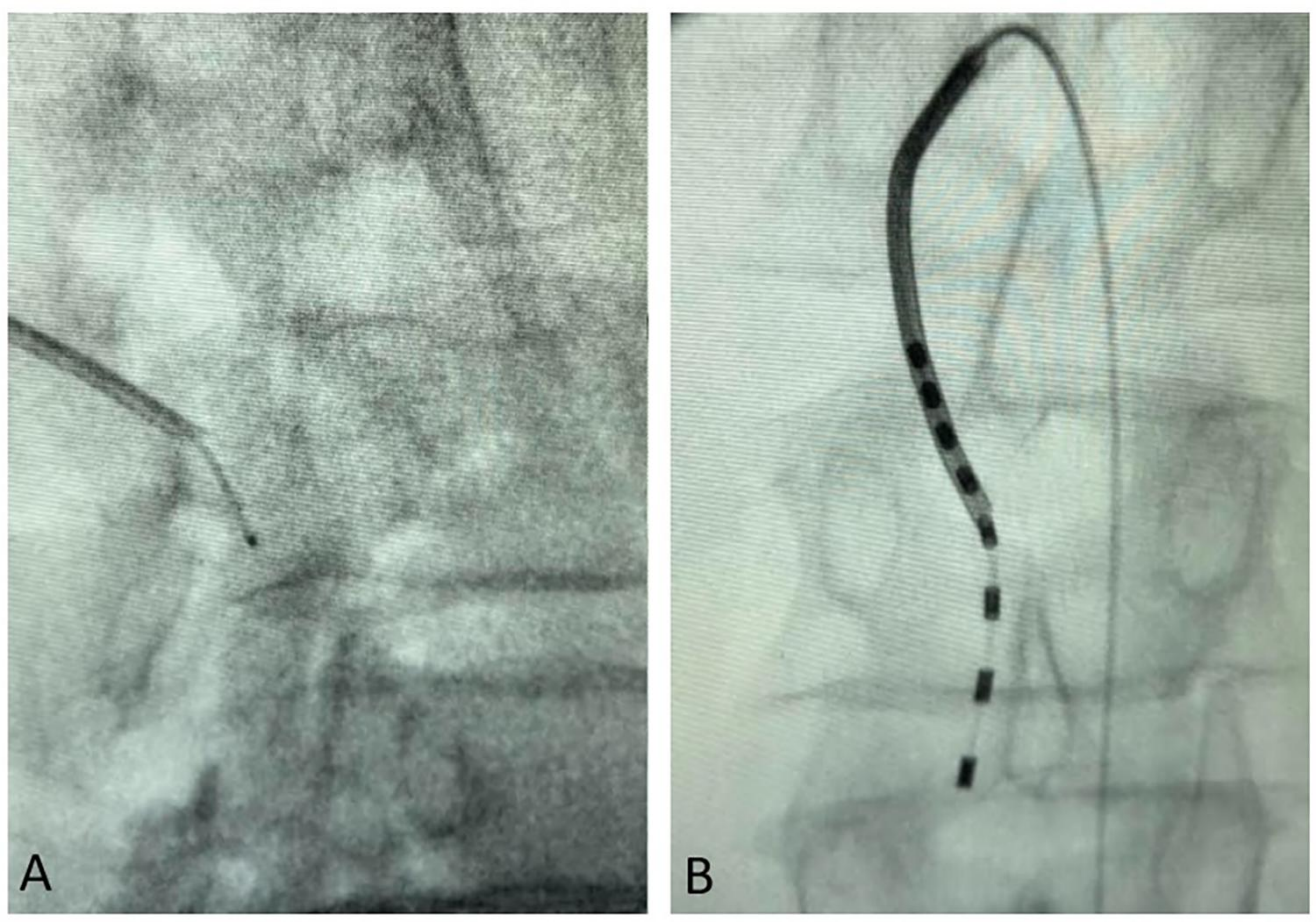
The lead blank serves a dual purpose: it identifies the epidural space **(A)** and creates a path for subsequent SCS lead placement **(B)**, analogous to drilling a pilot hole.

The SCS trial was performed in April of 2025. Retrograde placement of a sacral lead was first attempted at the L3-4 level from the left, using an Epimed 14 G needle bent opposite the bevel opening (Figure 1). Under contralateral oblique fluoroscopy, the needle was positioned along the ventral interlaminar line, and a Lead Blank (LB) was used to identify the epidural space (ES) (Figure 2A). After advancing the LB into the ES, a Nevro SCS lead was introduced (Figure 2B). Although the lead reached the L5-S1 level, resistance was encountered. After several unsuccessful attempts, the preloaded green curved stylet (0.014”) was replaced with a firmer blue curved stylet (0.016”) to facilitate advancement. Unfortunately, the firmer stylet perforated the SCS lead as it exited the needle bevel. To minimize the risk of accidental lead shredding, both the needle and the stylet-perforated lead were removed together.

**Figure 3:**
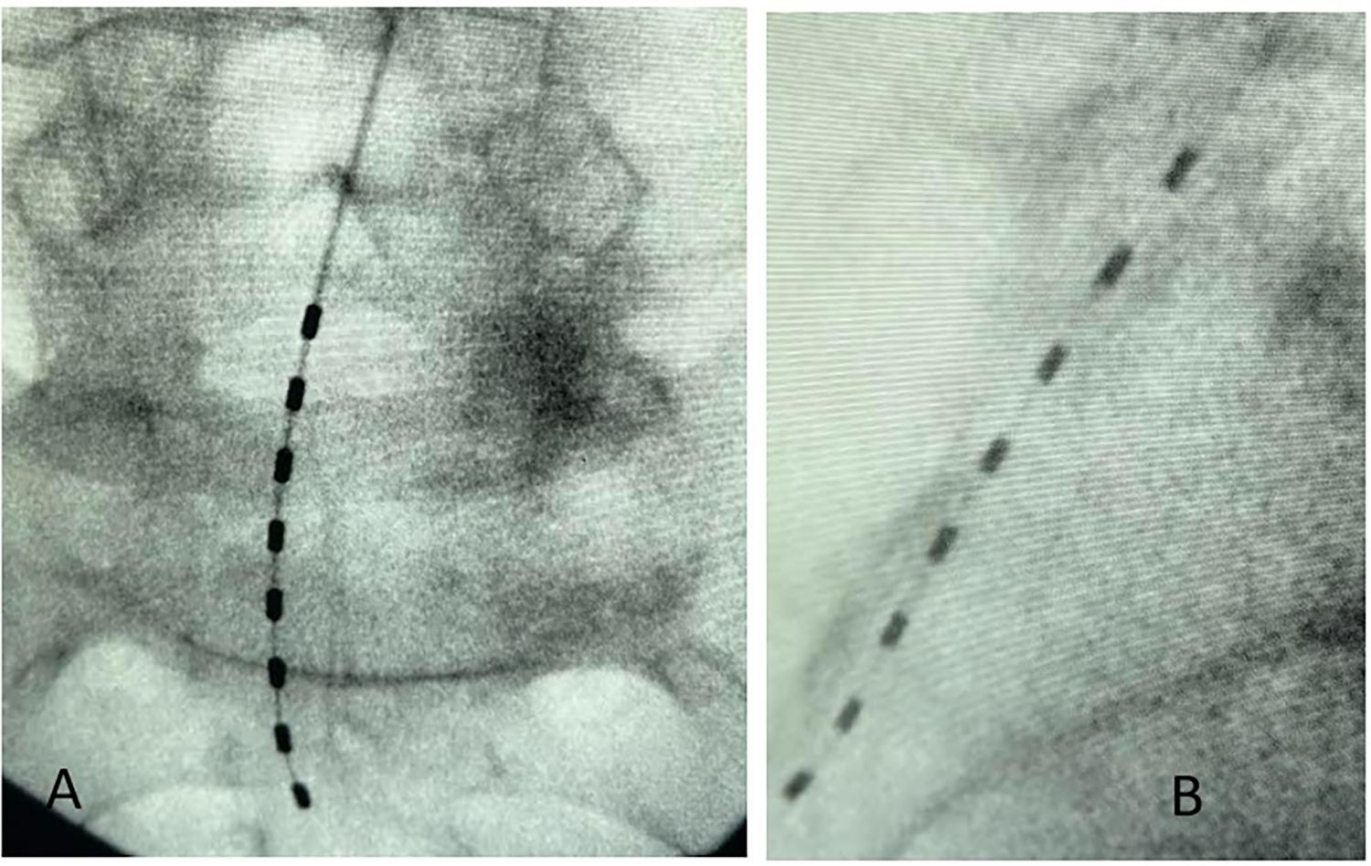
A retrograde SCS lead was positioned at the S3 level, with placement confirmed via anteroposterior **(A)** and lateral fluoroscopy **(B)**.

**Figure 4:**
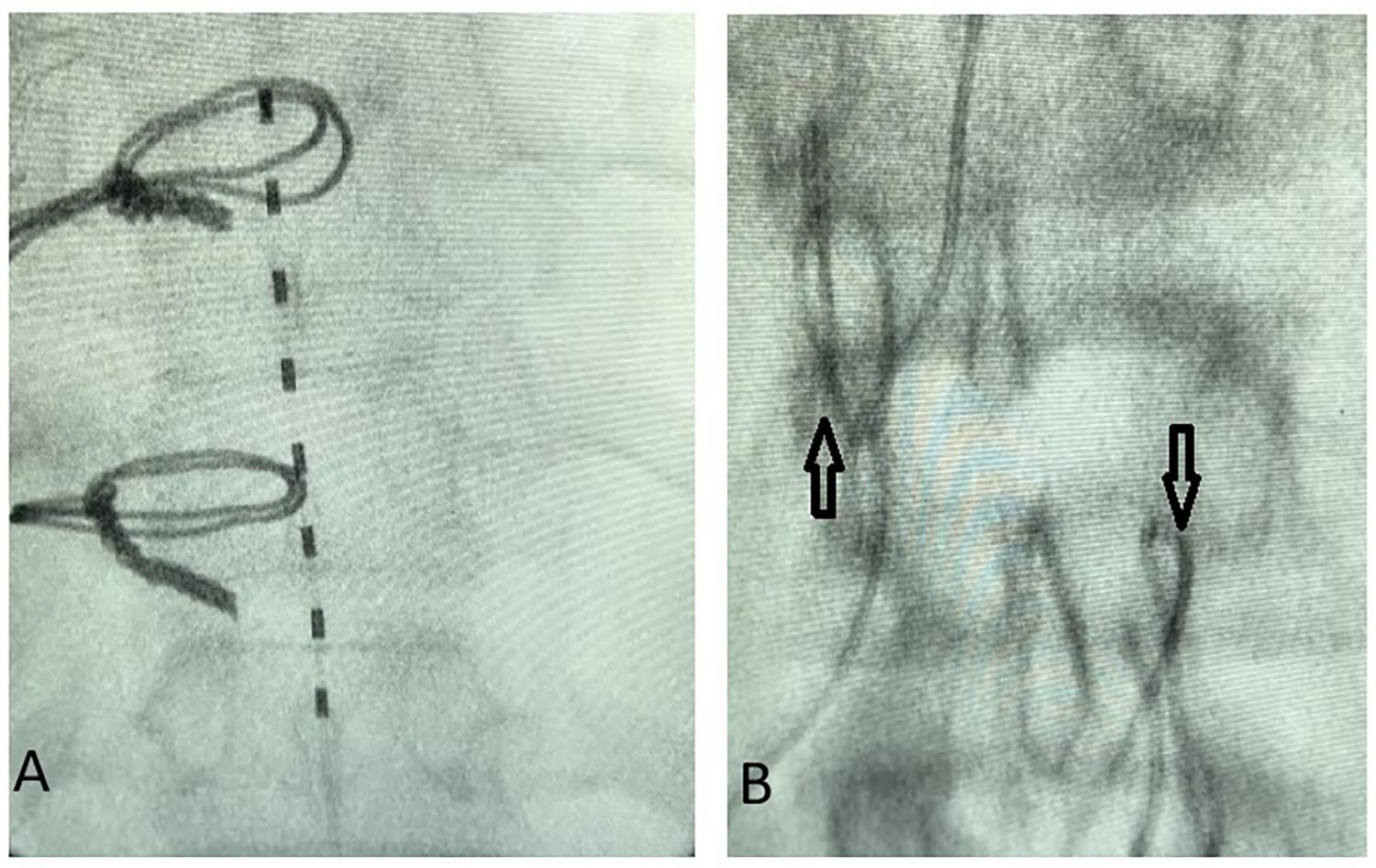
An anterograde SCS lead was secured by a subdermal security loop. The SCS lead tip is positioned at the upper margin of the T9 vertebral body (**A**). Sternal wires are visible following a previous thoracic aortic aneurysm repair. The upward arrow indicates the SSL for the thoracic lead; the downward arrow indicates the SSL for the sacral lead (**B**).

Retrograde placement at the L3-4 interlaminar space was then attempted from the right. Again, the Epimed needle was bent away from the bevel to facilitate insertion into the ES, and the LB was used to help identify the ES and advance the percutaneous lead. As with the initial attempt, the lead struggled to advance through the L5-S1 junction. On subsequent attempts, a firmer stylet was used without further complications, allowing smooth advancement to the S3 level. Proper lead positioning was confirmed via anteroposterior and lateral fluoroscopy (Figures 3A,B), and a subdermal security loop (SSL), an alternative to suturing, was created (Figure 4B) to prevent lead migration (7).

Anterograde SCS lead placement was performed without complication. The thoracic lead was placed at T9 (Figure 4A) and secured with an SSL (Figure 4B). Both leads were attached to the skin with Steri-Strips, Tegaderm, and fenestrated tape.

After the procedure, a Nevro representative tested both leads, educated the patient, and programmed two different stimulation frequency modes:

1. Tonic stimulation at 50 Hz, 250 *μ*s pulse width, with the amplitude gradually increased from 0.1 to 3.0 mA for the sacral lead.
2. High-frequency (HFX) stimulation at 10 kHz was used for the thoracic lead.

Within 24 h, the patient reported significant functional improvement, quantifying a 60% reduction in pelvic pain. Notably, she was able to maintain bathroom hygiene without discomfort for the first time in months. On the second day of the trial, she developed right hip pain concurrent with sacral tonic stimulation, resulting in a limp. She discontinued sacral stimulation after three days but continued HFX for the remainder of the seven-day trial. She also experienced bleeding at the procedural site, requiring two office visits for dressing changes within the first few days of the trial. Fluoroscopy confirmed no lead migration at the time of removal. Ultimately, she elected to proceed with HFX stimulation alone based on her trial experience, and the permanent SCS was implanted in September of 2025.

## 3 Discussion

The etiology of the pelvic pain experienced by our patient remains unclear, as such pain is an uncommon complication following emergency open thoracic aortic aneurysm repair. One potential cause might have been temporary ischemia in the pelvic region due to interrupted blood supply during aortic clamping. Regardless of the cause, severe pelvic pain is notoriously difficult to manage and significantly impairs quality of life. Despite extensive interventions—including procedures, physical therapy, psychotherapy, and multiple medications—significant improvement remained elusive. Although SCS is not a cure-all, it can improve the quality of life and functionality for patients experiencing such pain. Caudocephalad (anterograde) lead positioning targeting the thoracic spine, or cephalocaudal (retrograde) lead placement targeting the sacral spine, are preferred placements during SCS trials for pelvic pain.

Conventional anterograde thoracic SCS placements are effective in managing pelvic pain (8–11). Retrograde sacral lead placements are also recognized as effective (12–14), though they are performed less commonly due to technical challenges (15). The main difficulty lies not in epidural needle insertion inside the ES but in maneuvering the SCS lead within it. Bending the Epimed needle facilitated access to the interlaminar space during the challenging retrograde placement (Figures 1, 2B), while the LB aided both early ES identification and creation of a path for the SCS lead (Figures 2A,B). The coiled LB, previously included in all Nevro and Boston Scientific kits, has been supplied separately since October 2023 and is requested by us for each case. Although off-label and not currently considered part of the standard of care, epidural space recognition with the LB during SCS trials or permanent placements offers an alternative to the traditional loss of resistance technique. The LB's firmness enables perforation of the ligamentum flavum while the needle bevel remains within the ligament. Then, by following the needle tip's curve, the LB enters the ES, creating a path for the lead—analogous to drilling a pilot hole before inserting a screw. Interestingly, a similar technique was introduced in 2011 by Jie Zhu et al., who utilized the SCS's lead to identify the epidural space (16). The advantage of the LB over an SCS lead is that it is firmer and thinner, allowing it to perforate the ligamentum flavum more easily.

Navigating the lumbosacral joint presents another challenge, likely due to anatomical factors such as lumbosacral lordosis, sacral kyphosis, the sacral promontory, and age-related changes. Employing a firmer stylet can facilitate passage but may risk lead perforation, as occurred in this case, likely due to a kink in the lead as it passed through the needle bevel, which was pressing against the lumbar lamina. Carefully withdrawing both the needle and the perforated lead together can prevent lead shredding and the need for emergency removal of a retained foreign object.

Other strategies to improve lead navigation through the lumbosacral joint could include patient positioning techniques that minimize lumbar lordosis, such as the knee-to-chest or lateral decubitus positions. Using a wedge under the abdomen or employing reverse prone Trendelenburg positioning on the operating table may also assist with lead placement. An alternative to retrograde sacral placement is an anterograde approach via the sacral hiatus (17–19). This method is technically easier because it avoids the obstacles posed by retrograde placement. However, it also presents its own technical difficulties related to the caudal approach and securing the lead to prevent migration (17, 20).

There is research on the use of different stimulation modes simultaneously with one or two leads (combination therapy) (4–6), as well as on the use of combined anterograde (thoracic) and retrograde (sacral) SCS leads for pelvic pain (2, 3). However, to our knowledge, no published research has investigated both subjects together—specifically, anterograde-retrograde SCS placement with different stimulation modes applied to each lead. Furthermore, a literature search found no prior use of the term ‘combined bidirectional’ in this context, though it logically describes anterograde-retrograde placement, where leads are positioned in opposite directions. This differs from ‘combined unidirectional’ placement, in which both leads are inserted caudocephalad (e.g., a sacral lead via the sacral hiatus and a thoracic lead via the conventional approach).

Abdel-Aziz et al. (2) described a similar approach, which they termed the “combined sacral nerve roots stimulator and low thoracic spinal cord stimulator.” In their study, sacral nerve root stimulation was achieved via retrograde lead placement, whereas low thoracic SCS was achieved via anterograde lead placement. Paresthesia-type stimulation was applied at both sites. The authors reported immediate pain relief exceeding 50% by the fifth day of the trial, and all three patients subsequently underwent permanent SCS implantation.

The rationale for combined tonic and high-frequency stimulation at different spinal levels employed for our patient was to achieve cumulative therapeutic benefit. Tonic stimulation at the sacral spine targeted pelvic neuropathic pain, while simultaneous high-frequency stimulation at T9-T10 was intended to modulate pain signal transmission to the brain. Each lead was controlled by a separate external pulse generator, with the patient able to manage both stimulation types independently using the Nevro App on her iPhone (for HFX) and a remote control (for tonic stimulation).

The unexpected emergence of hip pain during the trial may be attributed to tonic stimulation of the lumbosacral plexus (L1–S4) and the sciatic nerve (L4–S3), both of which innervate the hip (21). Paresthesia and dysesthesia are recognized effects of tonic stimulation (22). Also, as the tonic stimulation intensity was gradually increased from 0.1 to 3 mA, higher amplitudes may produce more pronounced surface electromyography responses and trigger peripheral muscle contractions via motor fiber recruitment, thereby contributing to our patient's limp (23). Adjusting stimulation parameters or switching to alternative modes (sub-perceptional, high-frequency, or burst) may address this issue (24). Unfortunately, in this case, the complication influenced the patient's decision to forgo combined bidirectional stimulation in favor of thoracic HFX alone. Following her permanent thoracic HFX SCS placement, attempts to contact her were unsuccessful as she did not attend her scheduled follow-up visit.

## 4 Conclusion

While combined bidirectional spinal cord stimulation offers potential benefits for treating refractory neuropathic pelvic pain, its practical utility must be carefully weighed against the challenges of retrograde sacral lead placement, the risk of unexpected complications—as demonstrated in this case—and the limited evidence on long-term outcomes. Although combined bidirectional stimulation did not yield the anticipated results for our patient, it remains a consideration for future trials to guide optimal management.

## Data Availability

The raw data supporting the conclusions of this article will be made available by the authors, without undue reservation.
